# Transcriptome analysis of early pregnancy vitamin D status and spontaneous preterm birth

**DOI:** 10.1371/journal.pone.0227193

**Published:** 2020-01-29

**Authors:** Aishwarya P. Yadama, Hooman Mirzakhani, Thomas F. McElrath, Augusto A. Litonjua, Scott T. Weiss

**Affiliations:** 1 Channing Division of Network Medicine, Department of Medicine, Brigham and Women’s Hospital, and Harvard Medical School, Boston, MA, United States of America; 2 Division of Maternal Fetal Medicine, Department of Obstetrics and Gynecology, Brigham and Women’s Hospital, and Harvard Medical School, Boston MA, United States of America; 3 Golisano Children’s Hospital at Strong, University of Rochester Medical Center, Rochester, NY, United States of America; University of Southampton, UNITED KINGDOM

## Abstract

**Background:**

We conducted a literature review on the studies that investigated the relationship of preterm birth, including spontaneous preterm birth (sPTB), with vitamin D status. Overall, these studies demonstrated that the incidence of sPTB was associated with maternal vitamin D insufficiency in early pregnancy. However, the potential mechanisms and biological pathways are unknown.

**Objectives:**

To investigate early pregnancy gene expression signatures associated with both vitamin D insufficiency and sPTB. We further constructed a network of these gene signatures and identified the common biological pathways involved.

**Study design:**

We conducted peripheral blood transcriptome profiling at 10–18 weeks of gestation in a nested case-control cohort of 24 pregnant women who participated in the Vitamin D Antenatal Asthma Reduction Trial (VDAART). In this cohort, 8 women had spontaneous preterm delivery (21–32 weeks of gestation) and 17 women had vitamin D insufficiency (25-hydroxyvitamin D < 30 ng/mL). We separately identified vitamin D-associated and sPTB gene signatures at 10 to 18 weeks and replicated the overlapping signatures in the mid-pregnancy peripheral blood of an independent cohort with sPTB cases.

**Result:**

At 10–18 weeks of gestation, 146 differentially expressed genes (25 upregulated) were associated with both vitamin D insufficiency and sPTB in the discovery cohort (FDR < 0.05). Of these genes, 43 (25 upregulated) were replicated in the independent cohort of sPTB cases and controls with normal pregnancies (*P* < 0.05). Functional enrichment and network analyses of the replicated gene signatures suggested several highly connected nodes related to inflammatory and immune responses.

**Conclusions:**

Our gene expression study and network analyses suggest that the dysregulation of immune response pathways due to early pregnancy vitamin D insufficiency may contribute to the pathobiology of sPTB.

## Introduction

Preterm birth (PTB), defined as delivery occurring before 37 weeks of gestation, affects up to 10% of all pregnancies, of which, 45–50% are idiopathic or spontaneous [1, 2]. Spontaneous PTB (sPTB) is defined as commencement of labor with intact or prelabor rupture of membrane and birth before 37 weeks of gestation. While the risk factors and etiology of sPTB are still being investigated, several studies have investigated the association of vitamin D status with the incidence of sPTB. Several of these investigations provided evidence on the protective role of vitamin D during pregnancy in the prevention of both spontaneous and medically indicated PTB, however, a few found no association between vitamin D insufficiency and PTB [3–7]. These studies differ in methodology in that some investigated the impact of vitamin D supplementation, and some looked only at the association between vitamin D level (25-hydroxyvitamin D [25OHD]) during pregnancy and PTB. These studies also used varied definitions of vitamin D deficiency and sufficiency. More importantly, much of the available research on vitamin D and PTB considered vitamin D level at mid- or late pregnancy, while recent observations highlight the importance of the early vitamin D sufficiency in pregnancy and early vitamin D supplementation to rectify the insufficiency [8, 9].

As such, we conducted a literature review of studies investigated the relationship between vitamin D and PTB including sPTB [9–18]. In this work and considering the results from systematic review studies and meta-analysis of these prior investigations [11–14], we investigate the potential biological pathways related to early pregnancy vitamin D sufficiency status that might be related to sPTB specifically.

Gene expression profiling can be useful for identifying pathway genes that provide insight into understanding the molecular mechanisms responsible for sPTB at early pregnancy. Previous research has looked at early pregnancy peripheral blood gene expression in patients who had preterm deliveries, each finding a set of genes that can be explored further for their roles in PTB [19, 20]. Therefore, gene expression profiling could be employed as a helpful tool for exploring the biological pathways related to early pregnancy vitamin D status that may contribute to sPTB. We performed a nested case-control study in the Vitamin D Antenatal Asthma Reduction Trial (VDAART) to identify differentially expressed gene signatures associated with both vitamin D status and sPTB in early pregnancy. Systems biology approaches have revealed that disease-related genes distribute non-randomly in the protein-protein interaction network (interactome), thereby constructing a disease module [21, 22]. Accordingly, we examined the connectivity and modularity of the differentially expressed genes related to early pregnancy vitamin D status and sPTB to identify potential key drivers of sPTB module.

## Materials and methods

### VDAART design, participants, interventions, and oversight

VDAART (www.vdaart.com) is a randomized, double-blind, placebo-controlled clinical trial looking at the effect of vitamin D supplementation (4,000 IU vitamin D plus a multivitamin with 400 IU vitamin D daily) in comparison with a placebo (placebo pill plus a multivitamin containing 400 IU vitamin D) for pregnant women with a history of asthma or atopy. The trial aimed to determine whether vitamin D supplementation was associated with reduced incidence of asthma and recurrent wheeze in the participants’ children and to determine whether vitamin D supplementation reduced the incidence of adverse pregnancy outcomes such as preeclampsia. The details of the trial and inclusion criteria are published [23]. In brief, VDAART participants were pregnant women who were non-smoker and between 18 and 40 years old who were recruited at 10 and 18 weeks of gestation from 3 study centers in the United States: Boston University Medical Center in Boston, Massachusetts; Washington University in St. Louis, and Kaiser Permanente Southern California Region in San Diego, California [23]. VDAART was approved by the IRBs of the participating institutions (Washington University in St. Louis, Boston Medical Center, Kaiser Health Care San Diego) and Brigham and Women’s Hospital, and written consent was obtained from all participating pregnant women at their first enrollment visit. VDAART has been registered on ClinicalTrials.gov NCT00920621. This study is an ancillary and a nested-case control gene expression study in the VDAART cohort. Baseline maternal characteristics of the study subjects, those with sPTB and healthy controls, summarized in Table 1. The difference in proportions of study groups was compared using a Chi-Square test and two-tail P-values were reported. Student’s t-test was applied as appropriate.

**Table 1:** The VDAART subjects’ characteristics in the gene expression study.

	sPTB	Normal Pregnancy	P-Value
N	8	16	
Gestational age at enrollment (mean [SD])	14.80 (2.94)	14.63 (2.83)	0.892
Gestational age at delivery (mean [SD])	26.44 (3.57)	39.21 (0.93)	<0.001
Maternal Age (mean [SD])	28.73 (4.62)	28.22 (4.56)	0.800
Previous Pregnancies (%)			0.292
1st Pregnancy	5 (62.5)	5 (31.2)	
2nd Pregnancy	1 (12.5)	6 (37.5)	
3rd or more Pregnancies	2 (25.0)	5 (31.2)	
BMI at first appointment (mean [SD])	30.56 (4.82)	27.15 (7.02)	0.261
Study Site (%)			1
Boston	4 (50.0)	8 (50.0)	
San Diego	1 (12.5)	2 (12.5)	
St. Louis	3 (37.5)	6 (37.5)	
Maternal Race (%)			0.928
Asian	2 (25.0)	3 (18.8)	
Black or African American	3 (37.5)	7 (43.8)	
White	3 (37.5)	6 (37.5)	
Educational Level Completed (%)			0.337
College graduate	5 (62.5)	5 (31.2)	
Did not graduate from high school	0 (0.0)	4 (25.0)	
High school, technical school	1 (12.5)	3 (18.8)	
Junior college/some college	2 (25.0)	4 (25.0)	
Married (%)	4 (50.0)	7 (43.8)	1
Income (%)			0.472
Do not know/prefer not to answer	1 (12.5)	3 (18.8)	
Less than $50,000	3 (37.5)	9 (56.2)	
Over $50,000	4 (50.0)	4 (25.0)	
Maternal Asthma = Yes (%)	3 (37.5)	7 (43.8)	1
Maternal Eczema = Yes (%)	4 (50.0)	4 (25.0)	0.444
Maternal Allergic Rhinitis = Yes (%)	5 (62.5)	9 (56.2)	1
Vitamin D Insufficiency at 10–18 Weeks (%)	5 (62.5)	12 (75.0)	0.874
Treatment Arm	3 (37.5)	10 (62.5)	0.469

### Study subjects in discovery cohort (VDAART)

We selected PTB cases prior to 32 weeks of gestation who had spontaneous preterm labor (sPTB) and control subjects for gene expression analysis from the participants of VDAART, excluding pregnancy-induced hypertensive cases of preterm birth or preterm birth due to chorioamnionitis. In this study, we looked specifically at non-hypertensive and un-induced cases of PTB diagnosed with sPTB given that our previous research found an association between early-pregnancy vitamin D insufficiency and preeclampsia [24].

15 participants who had PTB with available and suitable RNA were considered for our gene expression study. Of these 15 participants, 7 samples were excluded if they had PTB due to hypertension during pregnancy, an induced delivery or a positive lab test for chorioamnionitis, preterm rupture of membrane, or delivery between 32–37 weeks of gestation or abruption. Our nested case-control group (N = 24) was comprised of 8 women who had sPTBs (21–32 weeks of gestation), and 2 matched controls per woman, for a total of 16 controls (Table 1). Control subjects were women with normal pregnancy and suitable RNA who were matched with sPTB cases on maternal age (within 5 years), race, and study site. In a post-matching comparison of controls, we found no significant difference in maternal age, race, or pregnancy gestational age among the 8 subjects with sPTB and the 16 controls. Overall, in the discovery (VDAART) cases and controls, 17 subjects (71%) had insufficient vitamin D status (<30 ng/mL, 5 with sPTB), and 7 (29%) had sufficient vitamin D status (≥30ng/mL, 3 with sPTB, Table 1).

### Measurement of Vitamin D (25OHD) in VDAART

Quantitative determination of 25-hydroxyvitamin D in the subjects’ plasma was assessed using the FDA approved, direct, competitive chemiluminescence immunoassay (CLIA) by the DiaSorin LIAISON 25-OH Vitamin D Total assay at the Channing Division of Network Medicine. This assay is co-specific for 25-hydroxyvitamin D3 and 25-hydroxyvitamin D2. The assay utilizes a specific antibody to 25-hydroxyvitamin D for coating magnetic particles (solid phase) and a vitamin D analogue, 22-carboxy-23,24,25,26,27-pentanorvitamin D3, linked to an isoluminol derivative. During the incubation, 25-hydroxyvitamin D is dissociated from its binding protein and competes with the isoluminol labeled analogue for binding sites on the antibody. After the incubation, the unbound material is removed with a wash cycle. Subsequently, the starter reagents are added, and a flash chemiluminescent reaction is initiated. The light signal is measured by a photomultiplier as relative light units (RLU) and is inversely proportional to the concentration of 25-hydroxyvitamin D present in calibrators, controls, or samples. The inter-and intra-assay Coefficients of Variability for this assay are 11.2% and 8.1%, respectively. For this study, Vitamin D insufficiency was defined at a 25(OH)D threshold of <30 ng/mL based on the Endocrine Society’s recommendations for health benefits2 and prior observation in the relationship of pregnancy vitamin D status and risk of adverse pregnancy outcomes [25] [26] [27].

### RNA isolation and microarray processing

Total RNA was isolated from whole blood using the QIAGEN PAXgene Blood RNA Kit according to the manufacturer’s protocol. The Ambion Globin Clear kit (Ambion^®^) is used to remove alpha and beta-globin mRNA from the sample. This procedure is done for whole blood samples to increase the sensitivity of gene expression assays, by improving the detection rate of expressed genes. The RNA was quantified using Nanodrop 8000 and checked for high integrity before the preparation of cDNA (first-strand synthesis). RNA concentration and purity of the sample were assessed using the Agilent 2100 Bioanalyzer, which estimates an RNA Integrity Number (RIN). Samples with RIN ≥ 8 were deemed acceptable and included in this analysis.

Gene expression was assessed using the Affymetrix Human Gene 1.0 ST Array. Biotinylated cRNA was prepared according to the manufacturer’s protocol, and hybridization was processed according to the protocol for the GeneChip Hybridization Control Kit at the Channing Division of Network Medicine, data coordinating center of the VDAART. As such using the isolated and processed RNA samples collected at 10 to 18 weeks of gestation, we created an expression set of 33,297 gene probes from 24 samples. The data discussed in this publication have been deposited in NCBI’s Gene Expression Omnibus and are accessible through GEO Series accession number GSE142974 available at https://www.ncbi.nlm.nih.gov/geo/query/acc.cgi?acc=GSE142974.

Quantiles of raw expression and principal components (PCs) across arrays were examined before and after background adjustment normalization and log_2_ transformation using iCheck package [28] and the results were compared by running the “QCReport” function in the R library’s “affyQCReport” [29]. We background adjusted, log_2_ transformed, and quantile normalized the arrays by applying the robust multiarray analysis (“rma”) function in R BioConductor’s “affy” library [30]. Probes were annotated using the annotation package “hugene10sttranscriptcluster.db” available on Bioconductor [31]. We limited the expression set to the annotated probes for autosomal chromosomes. Then, we applied the interquartile range (IQR) filter from R Bioconductor “genefilter” package [32] to remove the expressions with variance less than 20% within arrays and accounted for sources of expression heterogeneity and confounders (i.e., batch effect) using Surrogate Variable Analysis (SVA) using the “sva package” from the Bioconductor [33]. With the finalized expression set of 15,222 probes and 24 samples (8 with sPTB), we implemented the rank prod method for identifying differentially expressed genes by using R Bioconductor package “RankProd”[34]. All statistical analyses were performed using R version 3.6.0 [35].

### Replication cohort

To replicate the differentially expressed genes identified in the discovery cohort, we used a Gene Expression Omnibus (GEO) dataset that contained peripheral blood gene expression profiles from a cohort of pregnant women with and without sPTB at early and late pregnancy (17–23 and 27–33 weeks of gestation, respectively). The cohort included 51 cases with sPTB<37 weeks (10 cases had sPTB<32 weeks) matched with 114 controls who had normal pregnancies in the All Our Babies cohort in Calgary, Canada (N = 1878) [20]. The peripheral blood transcriptomes in this cohort were profiled using Affymetrix Human Gene 2.1 ST Array. We used the gene expressions from 17–23 weeks of gestation for the replication of our gene signatures and accessed the raw intensity files (.CEL) on GEO (https://www.ncbi.nlm.nih.gov/geo/query/acc.cgi?acc=GSE59491). The pre-processing of the CEL files, included background adjustment normalization and log_2_ transformation, were carried out using the RMA algorithm in the R Bioconductor “affy” library. Similar to the discovery dataset and after the quality control, we limited the replication expression set to the annotated probes for autosomal chromosomes with values of IQR including 80% of the probe expressions and accounted for sources of expression heterogeneity and confounders using SVA. The resulting expression set used for replication consisted of 53617 probes and 165 subjects (51 with sPTB).

### Gene expression analysis

**Fig 1:**
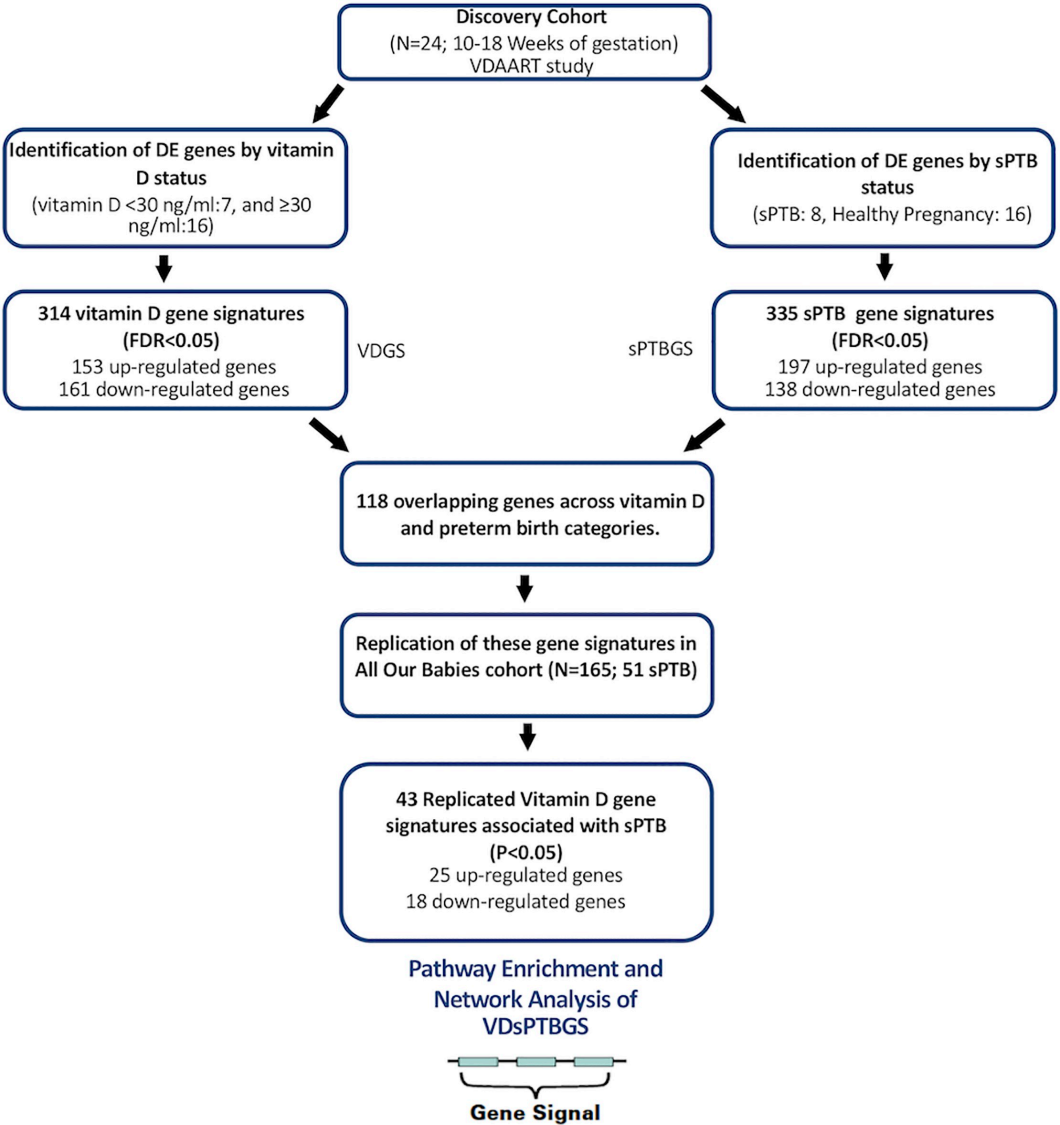
Gene expression study flowchart. Summary of analysis approach and findings on gene expression analysis through the discovery and replication stages.

R Bioconductor package “RankProd 2.0” [34] was used to perform differential expression analysis following quality control and adjustment for expression heterogeneity. “RankProd” is a non-parametric method that implements the Rank Product (RP) method for identifying genes that are consistently upregulated (or downregulated) in a number of replicated experiments. The advantage of the RP method is the non-parametric statistic which allows for increased performance in the case small-sample size and heterogeneity of samples [34, 36]. After running the RP differential expression method on the expression dataset from our discovery cohort, we obtained two set of differentially expression gene lists (FDR < 0.05) at 10–18 weeks of gestations: one consisting of genes associated with sPTB, and the other consisting of genes associated with vitamin D insufficiency (< 30 ng/mL). The overlapping genes of the two lists were determined and analyzed for differential expression in the replication cohort. We matched the probes in the discovery (VDDART) and replication (sPTB GEO) expression datasets by converting the platform-specific probe-ID to Entrez Gene ID [37]. The replicated gene signatures (*P*-value < 0.05) were considered for literature curation and database annotation in association with sPTB using MetaCore from Clarivate Analytics, GeneCards [38], and dbPTB [39] biological pathway enrichment, and network analyses. Fig 1 depicts an overview of our approach and a summary of the results at each step.

### Biological pathways of differentially expressed genes and their interaction in the interactome

We conducted functional enrichment of the replicated differentially expressed genes using gProfiler [40], which identifies a list of enriched functional terms from the Gene Ontology (GO) and other biological databases, ranked in order of statistical significance (FDR < 0.05). Further, we explored the connectivity and physical interaction of the replicated gene signatures by mapping them onto the human protein-protein interaction (PPI) network (interactome) using R Bioconductor package “STRINGdb” [41], which is the R interface for the STRING PPI database. We used molecular interactions with confidence score of > 0.4 demonstrating at least 50% confidence in the proposed interactions [41].The mapped gene set made up our sPTB module associated with vitamin D if demonstrated local enrichment in the PPI. To link the connected and disconnected components of the sPTB module, we looked at neighboring genes of the two submodules in the interactome and whether VDR gene is connected to the sPTB module through the direct interacting genes in its neighborhood, specifically IL-10, IL-8 and IL-6, which have immunologic roles in PTB [42].

## Results

### Early pregnancy vitamin D status modulates the expression of sPTB transcriptome signatures

In the discovery cohort, 314 genes (153 upregulated) had differential expression in the peripheral blood of women with vitamin D insufficiency (25OHD levels < 30 ng/mL) relative to those with sufficiency (25OHD levels ≥ 30 ng/mL) (FDR<0.05) at 10–18 weeks of gestation. Pregnant women who developed sPTB had 335 genes (197 upregulated) that were differentially expressed relative to those of controls at 10–18 weeks of gestation (FDR < 0.05, Fig 1, Table A in S1 File). The intersection of the vitamin D gene signatures and the sPTB gene signatures returned 118 overlapping genes with the same direction in expressions under each signature set. Of these overlapping gene signatures, 43 genes were differentially expressed (25 upregulated) in the peripheral blood of women with sPTB relative to those with normal pregnancy in the replication cohort (P<0.05; Table A in S1 File; Fig 1). Among these replicated genes, 31 genes (31/43[72.1%]) were previously reported in association with sPTB; 8 were found in a database search and 13 by manual curation through literature review (Table A in S1 File). Of these genes that have previously been reported in association with sPTB, we identified *MMP8*, *HLA-DQB1*, *IFI44L*, and several others in our curated list of 43 genes. Of those genes that have not been previously reported in association with sPTB (31/43[72.1%]), we identified *CLEC12A*, *CLEC12B*, *IFIT1B*, *KIAA1324* among others. The full gene list with annotations is reported in S1 File, Table A.

### Biological pathways of differentially expressed genes and their interaction in the interactome

In the Gene Ontology (GO) enrichment analysis 36 out 43 replicated gene signatures (83.72%) were enriched in several GO terms of immunologic functions including immune system process (N = 28) and response(N = 22), innate immune response (N = 12), ITGA2B-ITGB3 complex (N = 2), and leukocyte mediated immunity (N = 16, all corrected *P* < 0.05; Table B in S1 File).

**Fig 2:**
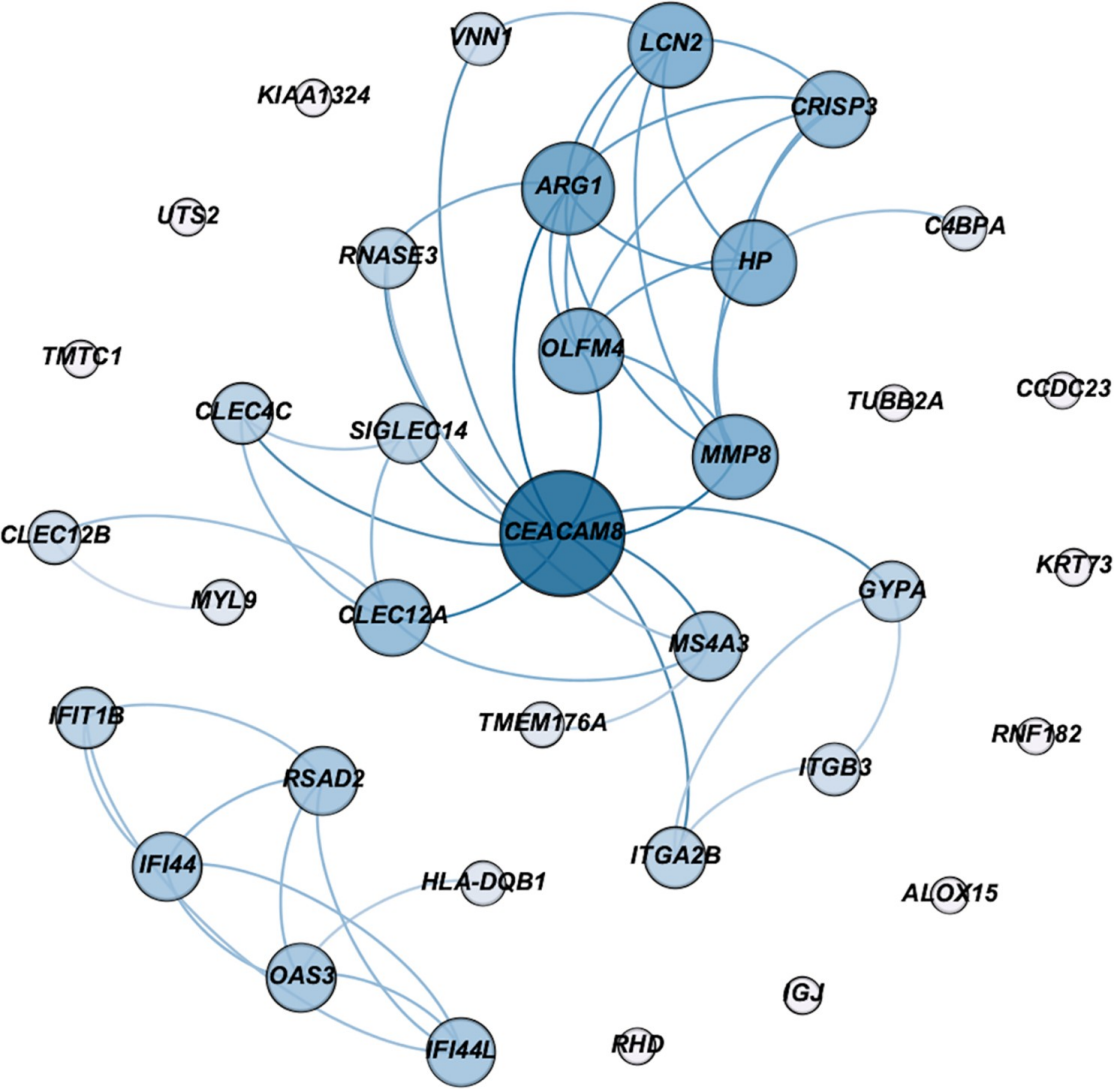
sPTB module constructed from 36 mapped replicated gene signatures with a large connected component (LCC, N = 20), and a small connected component (SCC, N = 6) as determined by evidence on the direct interaction in the interactome. The size of the nodes demonstrates the degree of connectivity and the darker blue gradient represents a higher betweenness centrality. GO enriched pathways and functional annotation are provided in S1 File, Table B.

**Fig 3:**
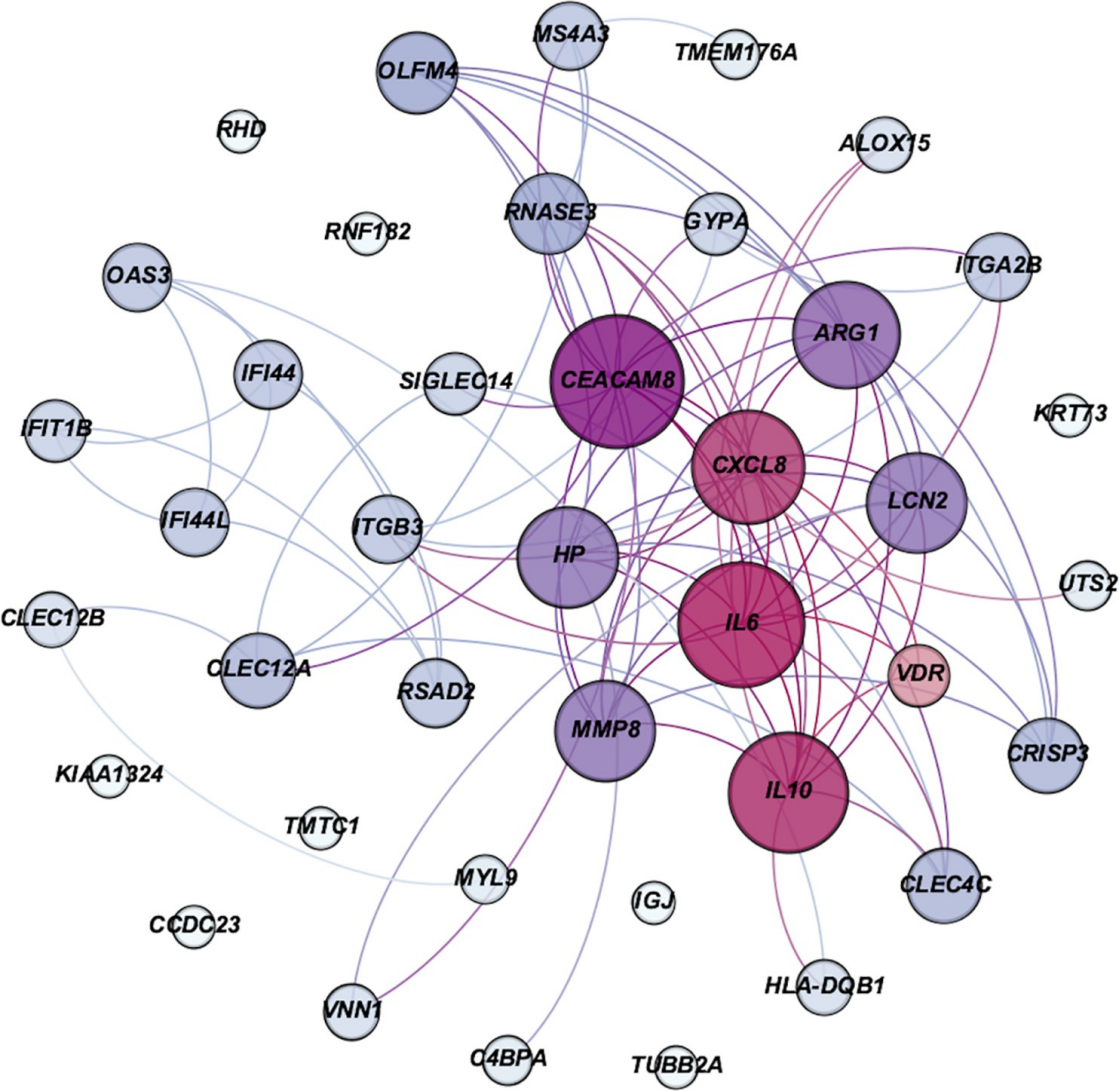
sPTB module with the inclusion of key neighboring genes associated with sPTB (IL10, IL8, IL6), showing a larger large connected component of sPTB module (expanded LCC, N = 32) and in connectivity with vitamin D receptor (VDR). The size of the nodes demonstrates the degree of connectivity and the darker purple gradient represents a higher betweenness centrality.

Of 43 replicated differentially expressed genes, 36 (83.72%) were mapped onto the PPI with local enrichment (*P* < 0.001) and constructed the sPTB module (Fig 2). 20 genes in the sPTB module had direct interactions in the PPI and constructed the largest connected component (LCC) of the sPTB module. Functional enrichment of the LCC in the sPTB module can be found in S1 File, Table C. A submodule in the sPTB module consisting of 6 genes constructed the small connected component (SCC, Fig 2). Among the 26 genes in these two submodules (LCC and SCC) *CEACAM8*, *OLFM4*, *IF44L*, *RSAD2*, *GYPA*, *ITGB3*, *MMP8*, and *OAS3* showed high degree of connectivity in their corresponding submodule and all were previously reported to be associated with sPTB (Fig 2, Table B in S1 File). VDR gene was connected to sPTB module through neighboring genes of *IL-10*, *IL8 (CXCL8)*, and *IL-6*. The addition of these 4 genes to the sPTB module, the size of LCC was increased from 20 to 32 genes (Fig 3). Several genes exhibited a high degree of betweenness, defined as the extent to which a gene lies on the shortest path between two other genes. Genes with a high betweenness centrality and connectivity degree can be leverage points in a network system due to their control over the passage of information between network components [42]. *IL10*, *IL8 (CXCL8)*, *IL6*, *CEACAM8*, *HP*, *MMP8* and *ARG1* were among the module of sPTB-associated genes that exhibited the highest betweenness centrality and connectivity degree (Fig 3).

## Discussion

This transcriptome analysis is founded on the large body of research showing an association between maternal vitamin D status and risk of PTB, including sPTB, concluded from meta-analysis and systematic review of several observational studies [11, 12, 14, 44]. A summary of the studies investigating the relationship between vitamin D and PTB can be found in Table 2.

**Table 2:** Literature curation of the association between vitamin D and preterm birth.

Journal, Authors, Year	Population	Design	Vitamin D Metric	Relevant Outcome Measured	Key Findings and Effect Estimate (95% CI)
Obstetrics and Gynecology; Bodnar et al. 2015[9]	N = 2327 (1126 Preterm)	Case-Cohort	Three cutoffs for analysis: <15.7 ng/mL, 15.7–23.6ng/mL, and ≥23.6 ng/mL	sPTB <37 weeks	-Mothers with 25(OH)D<15.7 ng/mL had a significant risk of sPTB at OR = 1.8; 95% CI (1.2–2.7) compared with those with 25(OH)D≥23.6 ng/mL
American Journal of Obstetrics and Gynecology; Dziadosz et al. 2014 [15]	N = 750 (67 Preterm)	Retrospective	Two cutoffs for analysis: <32 ng/ml and <20ng/ml	PTB<37 week	-25(OH)D deficiency in mothers increased risk of PTB at OR = 2.47; 95% CI (1.449–4.219)
Journal of Steroid Biochemistry and Molecular Biology; Wagner et al. 2016[45]	N = 509 (50 Preterm)	Post Hoc analysis of two combined RCTs	Three cutoffs for analysis: ≥20 ng/ml, 20–40 ng/ml, ≥40 ng/ml	PTB <37 Weeks	-Mothers with 25(OH)D ≥ 40 ng/ml had lower risk of PTB at OR = 0.41; 95% CI (0.20,0.86) as compared to those with 25(OH)D ≤20 ng/mL
The Journal of Nutrition; Tabatabaei et al. 2017[10]	N = 480 (120 PTB, 98 sPTB)	Case-Control	Three cutoffs for analysis: <15.7 ng/mL, 15.7–23.6ng/mL, and ≥23.6 ng/mL	PTB <37 weeks, sPTB<37 weeks	-Ethnic minority participants with 25(OH)D of 9.43 nm/L where at a higher risk of PTB than those with a concentration of 23.6 nm/L at OR = 4.05; 95% CI (1.16, 14.12). ' -There was no such association when considering only sPTB.
PLOS ONE; McDonnell et al 2017[17]	N = 1064 (139 PTB)	Case-Control	Four cutoffs for analysis: <20 ng/mL, 20 to <30 ng/mL, 30 to <40 ng/mL and ≥40 ng/mL	PTB <37 weeks	-Mothers with 25(OH)D ≥40 ng/ml were at a lower risk of PTB at OR = 0.42; 95% CI (0.2–0.89) compared to those with 25(OH)D <20 ng/ml
International Journal of Clinical Pathology; Zhu et al. 2015 [18]	N = 821 (180 PTB)	Prospective	Three cutoffs for analysis: <15.7 ng/mL, 15.7–23.6ng/mL, and ≥23.6 ng/mL	Very preterm: ≤31 weeks; Mildly Preterm: 32–37 weeks; term: >37 weeks of gestation	-25(OH)D deficiency occurred in 63.04% of pregnant women in very preterm group, compared to 36.61% in in-term group. -There was significant difference between the 25(OH)D level of the very preterm group and the other two groups (very preterm vs mildly preterm P<0.01, very preterm vs in-term group P<0.001) -No significant difference between the mildly preterm and in-term group (P = 0.47)
American Journal of Perinatology; Baker et al. 2011[46]	N = 4225 (40 sPTB)	Nested Case-Control	One cutoff for analysis: <15.7 ng/mL	sPTB ≥23 and ≤34 weeks' gestation	-Maternal 25(OH)D <15.7 ng/mL was not associated with sPTB in both adjusted and unadjusted models. Adjusted or OR = 0.82; 95% CI (0.19 to 3.57).
PLOS ONE; Flood-Nichols et al. 2015[47]	N = 235 (10 sPTB)	Retrospective	One cutoff for analysis: ≥30 ng/ml	sPTB <37 weeks	- No association between mothers with 25(OH)D ≥30 ng/ml and incidence of spontaneous preterm delivery with OR = 0.78; 95% CI (0.17–3.55)
British Journal of Obstetrics and Gynaecology; Rodriguez et al. 2014[6]	N = 2358 (108 PTB)	Prospective	Three cutoffs for analysis: <20 ng/mL, 20–29 ng/mL, ≥30 ng/mL	PTB <37 weeks	-There was no association between PTB and 25(OH)D levels at each cutoff. For 25(OH)D ≥30 ng/ml, OR = 1.08; 95% CI (0.75,1.67)
American Journal of Clinical Nutrition; Schneuer et al. 2014[7]	N = 5109 (388 PTB, 217 sPTB	Case-Control	Five cutoffs for analysis: <7.86, <11.8, 11.63–15.7, 15.7–23.6, >23.6	sPTB<37 Weeks All PTB<37 Weeks	-Mothers who had low 25(OH)D had a predisposition towards increased risk of sPTB than mothers who had normal levels of 25(OH)D at borderline significance (P = 0.09). -sPTB: Mothers with 25(OH)D<7.86 ng/mL had an adjusted OR = 1.47; 95% CI (0.77,2.82) relative to those with 25(OH)D from 15.7–23.6 ng/mL -PTB: Mothers with 25(OH)D<7.86 ng/L had an adjusted OR = 1.23; 95% CI (0.75,2.00) relative to those with 25(OH)D from 15.7–23.6 ng/mL
American Journal of Perinatology; Wetta et al. 2014[3]	N = 267 (90 sPTB)	Nested Case-Control	Three cutoffs for analysis: <15 ng/mL (deficient), < 30 ng/mL (insufficient), ≥30 (Normal)	sPTB<35 Weeks	-sPTB was not associated with either 25(OH)D insufficiency, with OR = 0.8; 95% CI (0.4, 1.4), or deficiency, with OR = 1.3; 95% CI (0.6, 3.0), at P-value = 0.62.
British Journal of Obstetrics and Gynaecology; Thorp et al. 2012[5]	N = 265 (131 PTB)	Nested Case-Control	One cutoff for analysis: <15.7 ng/mL	PTB<35 Weeks	-25(OH)D was not significantly correlated with preterm birth, with 25(OH)D concentrations ≥15.7 ng/mL generating OR = 0.8; 95% CI (0.38, 1.69) relative to those with less than 15.7 ng/mL.
Medical Science Monitor Yang et al. 2016[4]	N = 138 (46 PTB)	Prospective	Four cutoffs for analysis: <10 ng/mL, 10 to 20 ng/mL, 20 to 30 ng/mL and ≥30 ng/mL	PTB<37 Weeks	-Compared to those individuals with ≥30 ng/mL 25(OH)D, pregnant women with lower vitamin D (< 30 ng/mL) did not have significantly increased PTB risk OR = 0.90; 95% CI (0.45, 1.23). Similar insignificance was found across all other 25(OH)D cutoffs.
Obstetrics & Gynecology; Bodnar et al. 2015[9]	N = 3453 (1126 PTB)	Case-cohort	Three cutoffs for analysis: <20 ng/mL, 20 to 30 ng/mL, and ≥30 ng/mL	sPTB and PTB<37 Weeks sPTB and PTB <34 Weeks	The incidence of PTB and sPTB among mothers with 25(OH)D levels <20, 20–30 declined significantly as vitamin D levels improved. Risk of sPTB at less than 37 weeks of gestation, PTB less than 37 weeks of gestation, or preterm birth at less than 34 weeks of gestation among mothers with serum 25-hydroxyvitamin D less than 50 nmol/L was 1.8- fold to 2.1-fold greater than mothers with serum 25-hydroxyvitamin D 75 nmol/L or greater (OR = 1.8; 95% CI (1.2–2.7); OR = 1.8; 95% CI (1.3–2.6); OR = and 2.1; 95% CI (1.3–3.6).
Clinical Endocrinology; Sablok et al. 2015 [48]	N = 160	Randomized Control Trial	Three cutoffs for analysis: <7.9 ng/mL (deficient), 7.9 to 15.7 ng/mL (insufficient), and >15.7 ng/mL (sufficient)	preterm labor (PTL)<37 weeks	In the supplement group (sufficient subjects received one dose of 60,000 IU, insufficient subjects received two doses of 120,000 IU, deficient subjects received four doses of 120,000 IU), 8.3% of the patients had PTL whereas in placebo group, 21.1% had PTL, OR = 2.6; 95% CI(1.21–5.58); P = 0.02

The combined results from these studies led us to explore further the underlying mechanism through which vitamin D deficiency is linked to the incidence of preterm birth. It is important to note that there is a lack of consensus in the literature on what constitutes vitamin D sufficiency and insufficiency, which may have contributed to the inconsistency of results. Furthermore, few randomized trials have examined the effect of vitamin D supplementation on pregnancy outcomes such as PTB [14,49]. A systematic review of vitamin D supplementation and levels concerning birth outcomes indicated that such trials have small sample sizes and low dosage amounts of vitamin D [49]. No other study, to our knowledge, has explored the underlying biological mechanisms and gene signature of sPTB in relation to early pregnancy vitamin D status.

Our gene expression study returned a set of connected gene signatures related to both maternal vitamin D and sPTB. This network illuminated the connectivity of our differentially expressed genes to known vitamin D-signaling pathways and immunoinflammatory responses, thereby indicating a potential functional role of vitamin D on genes associated with sPTB. We further analyzed this network by measuring betweenness centrality for each node, or the number of shortest paths passing through a node, as well as its connectivity degree. This measurement depicted which genes in the network were the most responsible for monitoring communication between other genes in the network [22, 43]. The replicated genes we identified through our differential expression analysis were enriched in biological pathways related to maternal systemic changes in immune (innate and adaptive) and inflammatory responses (Fig 2). This result was particularly interesting due to the previously established ability of vitamin D to modulate innate and adaptive immune response [50].

Three neighboring genes that exhibited high levels of betweenness centrality and connectivity were *IL-6*, *IL-8* (*CXCL8*), and *IL10* which all are pro-inflammatory cytokines that previous research has identified as potential biomarkers for preterm birth (Fig 3). Previous research has shown that concentrations of maternal serum IL-6 and IL-8 levels are significantly higher in women who experienced preterm labor [51, 52]. Additionally, alterations in levels of anti-inflammatory cytokine Interleukin 10 (IL-10), a regulator of immune responses, have been linked pregnancy-related pathologic conditions such as preterm labor [53]. IL-10 reportedly suppresses the production of pro-inflammatory cytokines, and previous studies present evidence that IL-10 mRNA and protein are upregulated in gestational tissues in normal pregnancies [53]. VDR was linked to the sPTB module and it’s LCC through the neighboring 3 interleukin (IL) genes (Fig 3). This finding is in agreement with a prior study reporting that treatment of epithelial cells with 1,25(OH)_2_D_3_, a VDR agonist modulates IL-6 and IL-8 protein secretion [54]. The modulation of anti-inflammatory and pro-inflammatory cytokines is highly influential in the preparation of the placenta and pregnancy outcome. The down-regulation of *IL-6* and *IL-8* by 1,25(OH) _2_D_3_, would slow down cervical ripening and allow for the expression of anti-inflammatory cytokine IL-10 [51], one of the key nodes in our expanded sPTB module. Another notable biological process depicted in our network was the up-regulation of *MMP8*, metalloproteinase-8. A relevant scholarship has identified elevated levels of MMP8 in amniotic fluid is predictive of preterm delivery, finding that as many as 42% of deliveries before 32 weeks of gestation are associated with an elevated MMP-8 level [55]. The upregulation of these immune-related proteins and their proximity to the VDR in the interactome provide insight into the mechanisms of how vitamin D sufficient at early pregnancy might reduce the risk of sPTB.

A limitation of our gene expression study was the small sample size. We were able to identify only 8 sPTB cases (<32 weeks of gestation) who provided samples and had suitable RNA for transcriptome profiling and had the criteria for inclusion. This limitation and potential heterogeneity among the subjects might have affected the number of genes we were able to identify in our gene expression study at both discovery and replication stages which may have affected the size of sPTB module and comprehensiveness of the gene-gene interactions. The novel nature of our study made it difficult to find a replication cohort with both sPTB and vitamin D status, so we replicated subset of sPTB gene signatures that were also associated with vitamin D status in the discovery phase in an independent dataset according to the sPTB status. Similar to our discovery cohort, the sPTB cases in the replication cohort had a transcriptome profile from earlier pregnancy cohort; however, they had mostly sPTB at 32–37 weeks of gestation (N = 51, 10 cases with sPTB < 32 weeks), while our cases had sPTB < 32 weeks of gestation.

The identification of a network that represents an overlapping gene module between sPTB and vitamin D gene signatures, as well as the reduced risk of sPTB as a result of vitamin D sufficiency based on collective published evidence justify the necessity for further research on this association with a larger sample size. One consideration for such studies should be the vitamin supplementation dose. In the VDAART, only 74% of the subjects who received the 4,000 IU dose of vitamin D_3_ had a sufficient level of serum vitamin D (≥ 30 ng/mL) at 32 to 38 weeks of gestation, as compared to 82% at 1 month prior to delivery in an NICHD trial on a similar dose [56]. The results of these 2 studies highlight the latest emerging evidence that higher supplementation doses (2,000–6,000 IU/day) than the current recommendation of 400 to 600 IU/day, particularly in the absence of adequate sun exposure, might be necessary to support sufficient levels throughout pregnancy. Furthermore, we recommend pre-pregnancy, individualized doses for women who have a predisposition for vitamin D deficiency and/or a risk of non-adherence to supplementation. These recommendations need to be definitively tested in a pre-pregnancy clinical trial in a population of women at high risk of sPTB. Finally, we believe that follow-up studies should include a differential gene expression analysis of mothers with sPTB before 37 weeks of pregnancy.

In conclusion, the peripheral blood gene expression patterns of women who had sPTB show immune gene activation at 10 to 18 weeks, suggesting the importance of vitamin D sufficiency in early pregnancy. With further knowledge about the pathways underlying sPTB, we hope to be able to identify modifiable risk factors affecting these biological pathways related to sPTB and predict which subjects are predisposed to PTB based on their vitamin D levels and those risk factors; to achieve this, a larger study looking at vitamin D levels and supplementation in early pregnancy as it relates to sPTB is necessary.

## Supporting information

S1 File Replicated gene signatures of common genes with differential expression between sPTB and Vitamin D status and their literature curation (N = 43, Table A), Gene Ontology (GO) enrichment analysis of the replicated gene signatures that were mapped to the protein-protein interaction network, i.e., sPTB module (N = 36, Table B), and GO enrichment analysis of the Largest Connected Component (LCC) of sPTB module (N = 20, Table C).
(DOCX)

## References

[1] Vogel JP, Chawanpaiboon S, Moller AB, Watananirun K, Bonet M, Lumbiganon P. The global epidemiology of preterm birth. Best Pract Res Clin Obstet Gynaecol. 2018 Epub 2018/05/22. 10.1016/j.bpobgyn.2018.04.003 . 29779863

[2] Borg F, Gravino G, Schembri-Wismayer P, Calleja-Agius J. Prediction of preterm birth. Minerva Ginecol. 2013;65(3):345–60. Epub 2013/05/22. . 23689178

[3] Wetta LA, Biggio JR, Cliver S, Abramovici A, Barnes S, Tita AT. Is midtrimester vitamin D status associated with spontaneous preterm birth and preeclampsia? Am J Perinatol. 2014;31(6):541–6. Epub 2013/09/12. 10.1055/s-0033-1356483 24022379 PMC4451220

[4] Yang L, Pan S, Zhou Y, Wang X, Qin A, Huang Y, et al The Correlation Between Serum Vitamin D Deficiency and Preterm Birth. Med Sci Monit. 2016;22:4401–5. Epub 2016/11/17. 10.12659/MSM.898117 27851719 PMC5117241

[5] Thorp JM, Camargo CA, McGee PL, Harper M, Klebanoff MA, Sorokin Y, et al Vitamin D status and recurrent preterm birth: a nested case-control study in high-risk women. BJOG. 2012;119(13):1617–23. Epub 2012/10/20. 10.1111/j.1471-0528.2012.03495.x 23078336 PMC3546544

[6] Rodriguez A, Garcia-Esteban R, Basterretxea M, Lertxundi A, Rodriguez-Bernal C, Iniguez C, et al Associations of maternal circulating 25-hydroxyvitamin D3 concentration with pregnancy and birth outcomes. BJOG. 2015;122(12):1695–704. Epub 2014/09/12. 10.1111/1471-0528.13074 . 25208685

[7] Schneuer FJ, Roberts CL, Guilbert C, Simpson JM, Algert CS, Khambalia AZ, et al Effects of maternal serum 25-hydroxyvitamin D concentrations in the first trimester on subsequent pregnancy outcomes in an Australian population. Am J Clin Nutr. 2014;99(2):287–95. Epub 2013/11/22. 10.3945/ajcn.113.065672 . 24257720

[8] Hollis BW, Wagner CL. New insights into the vitamin D requirements during pregnancy. Bone Res. 2017;5:17030 Epub 2017/09/05. 10.1038/boneres.2017.30 28868163 PMC5573964

[9] Bodnar LM, Platt RW, Simhan HN. Early-pregnancy vitamin D deficiency and risk of preterm birth subtypes. Obstet Gynecol. 2015;125(2):439–47. Epub 2015/01/09. 10.1097/AOG.0000000000000621 25569002 PMC4304969

[10] Tabatabaei N, Auger N, Herba CM, Wei S, Allard C, Fink GD, et al Maternal Vitamin D Insufficiency Early in Pregnancy Is Associated with Increased Risk of Preterm Birth in Ethnic Minority Women in Canada. J Nutr. 2017;147(6):1145–51. Epub 2017/04/21. 10.3945/jn.116.241216 . 28424259

[11] Amegah AK, Klevor MK, Wagner CL. Maternal vitamin D insufficiency and risk of adverse pregnancy and birth outcomes: A systematic review and meta-analysis of longitudinal studies. PLoS One. 2017;12(3):e0173605 Epub 2017/03/18. 10.1371/journal.pone.0173605 28306725 PMC5357015

[12] Qin LL, Lu FG, Yang SH, Xu HL, Luo BA. Does Maternal Vitamin D Deficiency Increase the Risk of Preterm Birth: A Meta-Analysis of Observational Studies. Nutrients. 2016;8(5). Epub 2016/05/24. 10.3390/nu8050301 27213444 PMC4882713

[13] Bi WG, Nuyt AM, Weiler H, Leduc L, Santamaria C, Wei SQ. Association Between Vitamin D Supplementation During Pregnancy and Offspring Growth, Morbidity, and Mortality: A Systematic Review and Meta-analysis. JAMA Pediatr. 2018;172(7):635–45. Epub 2018/05/31. 10.1001/jamapediatrics.2018.0302 29813153 PMC6137512

[14] Zhou SS, Tao YH, Huang K, Zhu BB, Tao FB. Vitamin D and risk of preterm birth: Up-to-date meta-analysis of randomized controlled trials and observational studies. J Obstet Gynaecol Res. 2017;43(2):247–56. Epub 2017/02/06. 10.1111/jog.13239 . 28150405

[15] Dziadosz M, Parisi V, Warren W, Miller R. 802: Vitamin D deficiency in early gestation and rates of preterm birth: a retrospective cohort. American Journal of Obstetrics and Gynecology. 2014;210(1). 10.1016/j.ajog.2013.10.835

[16] Wagner CL, Baggerly C, McDonnell S, Baggerly KA, French CB, Baggerly L, et al Post-hoc analysis of vitamin D status and reduced risk of preterm birth in two vitamin D pregnancy cohorts compared with South Carolina March of Dimes 2009–2011 rates. J Steroid Biochem Mol Biol. 2016;155(Pt B):245–51. Epub 2015/11/12. 10.1016/j.jsbmb.2015.10.022 26554936 PMC5215876

[17] McDonnell SL, Baggerly KA, Baggerly CA, Aliano JL, French CB, Baggerly LL, et al Maternal 25(OH)D concentrations >/ = 40 ng/mL associated with 60% lower preterm birth risk among general obstetrical patients at an urban medical center. PLoS One. 2017;12(7):e0180483 Epub 2017/07/25. 10.1371/journal.pone.0180483 28738090 PMC5524288

[18] Zhu T, Liu TJ, Ge X, Kong J, Zhang LJ, Zhao Q. High prevalence of maternal vitamin D deficiency in preterm births in northeast China, Shenyang. Int J Clin Exp Pathol. 2015;8(2):1459–65. Epub 2015/05/15. 25973031 PMC4396334

[19] Enquobahrie DA, Williams MA, Qiu C, Muhie SY, Slentz-Kesler K, Ge Z, et al Early pregnancy peripheral blood gene expression and risk of preterm delivery: a nested case control study. BMC Pregnancy Childbirth. 2009;9:56 Epub 2009/12/17. 10.1186/1471-2393-9-56 20003277 PMC2799378

[20] Heng YJ, Pennell CE, Chua HN, Perkins JE, Lye SJ. Whole blood gene expression profile associated with spontaneous preterm birth in women with threatened preterm labor. PLoS One. 2014;9(5):e96901 Epub 2014/05/16. 10.1371/journal.pone.0096901 24828675 PMC4020779

[21] Goh KI, Cusick ME, Valle D, Childs B, Vidal M, Barabasi AL. The human disease network. Proc Natl Acad Sci U S A. 2007;104(21):8685–90. Epub 2007/05/16. 10.1073/pnas.0701361104 17502601 PMC1885563

[22] Menche J, Sharma A, Kitsak M, Ghiassian SD, Vidal M, Loscalzo J, et al Disease networks. Uncovering disease-disease relationships through the incomplete interactome. Science. 2015;347(6224):1257601 Epub 2015/02/24. 10.1126/science.1257601 25700523 PMC4435741

[23] Litonjua AA, Lange NE, Carey VJ, Brown S, Laranjo N, Harshfield BJ, et al The Vitamin D Antenatal Asthma Reduction Trial (VDAART): rationale, design, and methods of a randomized, controlled trial of vitamin D supplementation in pregnancy for the primary prevention of asthma and allergies in children. Contemp Clin Trials. 2014;38(1):37–50. Epub 2014/03/13. 10.1016/j.cct.2014.02.006 24614387 PMC4086903

[24] Mirzakhani H, Carey VJ, McElrath TF, Laranjo N, O'Connor G, Iverson RE, et al The Association of Maternal Asthma and Early Pregnancy Vitamin D with Risk of Preeclampsia: An Observation From Vitamin D Antenatal Asthma Reduction Trial (VDAART). J Allergy Clin Immunol Pract. 2018;6(2):600–8 e2. Epub 2017/09/20. 10.1016/j.jaip.2017.07.018 28923490 PMC5843492

[25] Holick MF, Binkley NC, Bischoff-Ferrari HA, Gordon CM, Hanley DA, Heaney RP, et al Evaluation, treatment, and prevention of vitamin D deficiency: an Endocrine Society clinical practice guideline. J Clin Endocrinol Metab. 2011;96(7):1911–30. Epub 2011/06/08. 10.1210/jc.2011-0385 . 21646368

[26] Mirzakhani H, Litonjua AA, McElrath TF, O'Connor G, Lee-Parritz A, Iverson R, et al Early pregnancy vitamin D status and risk of preeclampsia. J Clin Invest. 2016;126(12):4702–15. Epub 2016/11/15. 10.1172/JCI89031 27841759 PMC5127689

[27] Wei SQ. Vitamin D and pregnancy outcomes. Curr Opin Obstet Gynecol. 2014;26(6):438–47. Epub 2014/10/14. 10.1097/GCO.0000000000000117 . 25310531

[28] Weiliang Qiu BG, Christopher Anderson, Barbara Klanderman, Vincent Carey, Benjamin Raby. iCheck: QC Pipeline and Data Analysis Tools for High-Dimensional Illumina mRNA Expression Data R package. 1.14.0 ed2019.

[29] Parman C HC, Gentleman R. affyQCReport:QC Report Generation for affyBatch objects. R package. 1.64.0 ed2019.

[30] Gautier L, Cope L, Bolstad BM, Irizarry RA. affy—analysis of Affymetrix GeneChip data at the probe level. Bioinformatics. 2004;20(3):307–15. Epub 2004/02/13. 10.1093/bioinformatics/btg405 . 14960456

[31] JW M. hugene10sttranscriptcluster.db: Affymetrix hugene10 annotation data (chip hugene10sttranscriptcluster. R package. 8.7.0 ed2017.

[32] R. Gentleman VC, W. Huber, F. Hahne. genefilter: methods for filtering genes from high-throughput experiments. R package2019.

[33] Leek JT, Johnson WE, Parker HS, Jaffe AE, Storey JD. The sva package for removing batch effects and other unwanted variation in high-throughput experiments. Bioinformatics. 2012;28(6):882–3. Epub 2012/01/20. 10.1093/bioinformatics/bts034 22257669 PMC3307112

[34] Del Carratore F, Jankevics A, Eisinga R, Heskes T, Hong F, Breitling R. RankProd 2.0: a refactored bioconductor package for detecting differentially expressed features in molecular profiling datasets. Bioinformatics. 2017;33(17):2774–5. Epub 2017/05/10. 10.1093/bioinformatics/btx292 28481966 PMC5860065

[35] Team RC. R: A language and environment for statistical computing. Vienna, Austria: R Foundation for Statistical Computing; 2019.

[36] Breitling R, Herzyk P. Rank-based methods as a non-parametric alternative of the T-statistic for the analysis of biological microarray data. J Bioinform Comput Biol. 2005;3(5):1171–89. Epub 2005/11/10. 10.1142/s0219720005001442 . 16278953

[37] Allen JD, Wang S, Chen M, Girard L, Minna JD, Xie Y, et al Probe mapping across multiple microarray platforms. Brief Bioinform. 2012;13(5):547–54. Epub 2011/12/27. 10.1093/bib/bbr076 PubMed Central PMCID: PMC3431719. 22199380 PMC3431719

[38] Stelzer G, Rosen N, Plaschkes I, Zimmerman S, Twik M, Fishilevich S, et al The GeneCards Suite: From Gene Data Mining to Disease Genome Sequence Analyses. Curr Protoc Bioinformatics. 2016;54:1 30 1–1 3. Epub 2016/06/21. 10.1002/cpbi.5 . 27322403

[39] Uzun A, Laliberte A, Parker J, Andrew C, Winterrowd E, Sharma S, et al dbPTB: a database for preterm birth. Database (Oxford). 2012;2012:bar069 Epub 2012/02/11. 10.1093/database/bar069 22323062 PMC3275764

[40] Reimand J, Kull M, Peterson H, Hansen J, Vilo J. g:Profiler—a web-based toolset for functional profiling of gene lists from large-scale experiments. Nucleic Acids Res. 2007;35(Web Server issue):W193–200. Epub 2007/05/05. 10.1093/nar/gkm226 17478515 PMC1933153

[41] Szklarczyk D, Morris JH, Cook H, Kuhn M, Wyder S, Simonovic M, et al The STRING database in 2017: quality-controlled protein-protein association networks, made broadly accessible. Nucleic Acids Res. 2017;45(D1):D362–D8. Epub 2016/12/08. 10.1093/nar/gkw937 27924014 PMC5210637

[42] Peltier MR. Immunology of term and preterm labor. Reprod Biol Endocrinol. 2003;1:122 Epub 2003/12/04. 10.1186/1477-7827-1-122 14651749 PMC305338

[43] Koschutzki D, Schreiber F. Centrality analysis methods for biological networks and their application to gene regulatory networks. Gene Regul Syst Bio. 2008;2:193–201. Epub 2008/01/01. 10.4137/grsb.s702 19787083 PMC2733090

[44] Tous M, Villalobos M, Iglesias L, Fernandez-Barres S, Arija V. Vitamin D status during pregnancy and offspring outcomes: a systematic review and meta-analysis of observational studies. Eur J Clin Nutr. 2019 Epub 2019/01/27. 10.1038/s41430-018-0373-x . 30683894

[45] Wagner CL, Baggerly C, McDonnell SL, Baggerly L, Hamilton SA, Winkler J, et al Post-hoc comparison of vitamin D status at three timepoints during pregnancy demonstrates lower risk of preterm birth with higher vitamin D closer to delivery. J Steroid Biochem Mol Biol. 2015;148:256–60. Epub 2014/12/03. 10.1016/j.jsbmb.2014.11.013 25448734 PMC4415820

[46] Baker AM, Haeri S, Camargo CA, Jr., Stuebe AM, Boggess KA. A nested case-control study of first-trimester maternal vitamin D status and risk for spontaneous preterm birth. Am J Perinatol. 2011;28(9):667–72. Epub 2011/04/19. 10.1055/s-0031-1276731 21500145 PMC4372898

[47] Flood-Nichols SK, Tinnemore D, Huang RR, Napolitano PG, Ippolito DL. Vitamin D deficiency in early pregnancy. PLoS One. 2015;10(4):e0123763 Epub 2015/04/22. 10.1371/journal.pone.0123763 25898021 PMC4405493

[48] Sablok A, Batra A, Thariani K, Batra A, Bharti R, Aggarwal AR, et al Supplementation of vitamin D in pregnancy and its correlation with feto-maternal outcome. Clin Endocrinol (Oxf). 2015;83(4):536–41. Epub 2015/02/17. 10.1111/cen.12751 . 25683660

[49] Thorne-Lyman A, Fawzi WW. Vitamin D during pregnancy and maternal, neonatal and infant health outcomes: a systematic review and meta-analysis. Paediatr Perinat Epidemiol. 2012;26 Suppl 1:75–90. Epub 2012/07/07. 10.1111/j.1365-3016.2012.01283.x 22742603 PMC3843348

[50] Aranow C. Vitamin D and the immune system. J Investig Med. 2011;59(6):881–6. Epub 2011/04/30. 10.2310/JIM.0b013e31821b8755 21527855 PMC3166406

[51] Pandey M, Chauhan M, Awasthi S. Interplay of cytokines in preterm birth. Indian J Med Res. 2017;146(3):316–27. Epub 2018/01/23. 10.4103/ijmr.IJMR_1624_14 29355137 PMC5793465

[52] Shahshahan Z, Hashemi L. Maternal serum cytokines in the prediction of preterm labor and response to tocolytic therapy in preterm labor women. Adv Biomed Res. 2014;3:126 Epub 2014/06/21. 10.4103/2277-9175.133243 24949297 PMC4063106

[53] Mobini M, Mortazavi M, Nadi S, Zare-Bidaki M, Pourtalebi S, Arababadi MK. Significant roles played by interleukin-10 in outcome of pregnancy. Iran J Basic Med Sci. 2016;19(2):119–24. Epub 2016/04/16. 27081455 PMC4818358

[54] McNally P, Coughlan C, Bergsson G, Doyle M, Taggart C, Adorini L, et al Vitamin D receptor agonists inhibit pro-inflammatory cytokine production from the respiratory epithelium in cystic fibrosis. J Cyst Fibros. 2011;10(6):428–34. Epub 2011/07/26. 10.1016/j.jcf.2011.06.013 . 21784717

[55] Kim A, Lee ES, Shin JC, Kim HY. Identification of biomarkers for preterm delivery in mid-trimester amniotic fluid. Placenta. 2013;34(10):873–8. Epub 2013/08/21. 10.1016/j.placenta.2013.06.306 . 23953866

[56] Hollis BW, Johnson D, Hulsey TC, Ebeling M, Wagner CL. Vitamin D supplementation during pregnancy: double-blind, randomized clinical trial of safety and effectiveness. J Bone Miner Res. 2011;26(10):2341–57. Epub 2011/06/28. 10.1002/jbmr.463 21706518 PMC3183324

