# Supplementary material for: Transcriptome analysis of early pregnancy vitamin D status and spontaneous preterm birth
Source: PLoS One. 2020 Jan 29;15(1):e0227193. doi: 10.1371/journal.pone.0227193 (PMC6988958; doi:10.1371/journal.pone.0227193)
Supplement: S1 File — Replicated gene signatures of common genes with differential expression between sPTB and Vitamin D status and their literature curation (N = 43, Table A), Gene Ontology (GO) enrichment analysis of the replicated gene signatures that were mapped to the protein-protein interaction network, i.e., sPTB module (N = 36, Table B), and GO enrichment analysis of the Largest Connected Component (LCC) of sPTB module (N = 20, Table C). (DOCX) [file pone.0227193.s001.docx]

**Supplemental File 1 (S1 File)**

**Table A**

| **Gene SYMBOL** | **Gene Probe** | **Fold Chang (Control/sPTB)** | **Regulation** | **P-value** | **dbPTB Annotation** | **MetaCore/ GeneCards Annotation** | **Literature Curation** |
| --- | --- | --- | --- | --- | --- | --- | --- |
| ALOX15 | 8011680 | 1.324 | Up | 1.16E-05 | Not Curated | Curated | Not Reported |
| ARG1 | 8122058 | 1.218 | Up | 1.90E-04 | Not Curated | Curated | Not Reported |
| C4BPA | 7909318 | 1.624 | Up | 6.31E-07 | https://www.ncbi.nlm.nih.gov/pubmed/23591632 | Curated | https://www.ncbi.nlm.nih.gov/pmc/articles/PMC3868364/ |
| CEACAM8 | 8037222 | 1.137 | Up | 1.40E-04 | Not Curated | Not Curated | Not Reported |
| CLEC12A | 7953901 | 1.963 | Up | 2.71E-08 | Not Curated | Not Curated | Not Reported |
| CLEC12B | 7953914 | 2.178 | Up | 1.31E-07 | Not Curated | Not Curated | Not Reported |
| CLEC4C | 7960832 | 0.768 | Down | 4.43E-04 | Not Curated | Not Curated | Not Reported |
| CRISP3 | 8126905 | 1.093 | Up | 6.87E-07 | Not Curated | Curated | <https://www.ncbi.nlm.nih.gov/pubmed/26874297>; <https://www.ncbi.nlm.nih.gov/pubmed/29305255> |
| GYPA | 8102998 | 1.169 | Up | 2.07E-07 | Not Curated | Not Curated | http://dmd.aspetjournals.org/content/dmd/39/6/1000.full.pdf?with-ds=yes |
| HLA-DQB1 | 8125461 | 0.6841 | Down | 1.36E-04 | Not Curated | Curated | https://www.ncbi.nlm.nih.gov/pmc/articles/PMC3864772/ |
| HP | 7997188 | 1.205 | Up | 3.54E-08 | https://www.ncbi.nlm.nih.gov/pubmed/20003277 | Curated | Not Reported |
| IFI44 | 7902553 | 0.7741 | Down | 5.48E-06 | Not Curated | Curated | Not Reported |
| IFI44L | 7902541 | 0.7428 | Down | 9.45E-09 | Not Curated | Curated | https://www.ncbi.nlm.nih.gov/pmc/articles/PMC2852874/ |
| IFIT1B | 7929061 | 1.345 | Up | 4.82E-04 | Not Curated | Not Curated | Not Reported |
| IGKC | 8043468 | 1.344 | Up | 2.75E-04 | Not Curated | Not Curated | https://patents.google.com/patent/US20150355188 |
| ITGA2B | 8016044 | 1.206 | Up | 6.62E-06 | Not Curated | Curated | Not Reported |
| ITGB3 | 8007931 | 1.402 | Up | 2.85E-06 | Not Curated | Curated | http://amp.pharm.mssm.edu/Harmonizome/gene_set/Premature+Birth/CTD+Gene-Disease+Associations |
| JCHAIN | 8100827 | 1.726 | Up | 4.45E-04 | Not Curated | Not Curated | http://amp.pharm.mssm.edu/Harmonizome/gene_set/Premature+Birth/CTD+Gene-Disease+Associations |
| KIAA1324 | 7903592 | 0.7705 | Down | 1.10E-07 | Not Curated | Curated | Not Reported |
| KRT73 | 7963471 | 0.7894 | Down | 7.02E-05 | Not Curated | Not Curated | Not Reported |
| LCN2 | 8158167 | 1.148 | Up | 4.95E-06 | https://www.biorxiv.org/content/biorxiv/early/2018/06/29/358945.full.pdf | Curated | http://amp.pharm.mssm.edu/Harmonizome/gene_set/Premature+Birth/CTD+Gene-Disease +Associations |
| LILRA3 | 8039226 | 0.8072 | Down | 1.31E-04 | Not Curated | Not Curated | https://www.ncbi.nlm.nih.gov/pmc/articles/PMC5909855/ |
| LINC00328 | 8071049 | 0.7591 | Down | 4.17E-04 | Not Curated | Not Curated | Not Reported |
| LINC02520 | 8180245 | 1.047 | Up | 3.31E-05 | Not Curated | Not Curated | Not Reported |
| MMP8 | 7951246 | 0.9662 | Down | 4.70E-08 | https://www.ncbi.nlm.nih.gov/pubmed/19019335 | Not Curated | https://www.ncbi.nlm.nih.gov/pubmed/16170284 |
| MS4A3 | 7940216 | 0.8788 | Down | 1.79E-04 | https://www.ncbi.nlm.nih.gov/pubmed/20003277 | Not Curated | https://www.ncbi.nlm.nih.gov/pmc/articles/PMC2799378/ |
| MYL9 | 8062312 | 1.259 | Up | 3.52E-04 | https://www.ncbi.nlm.nih.gov/pmc/articles/PMC5112111/ | Curated | https://www.ncbi.nlm.nih.gov/pmc/articles/PMC5112111/ |
| OAS3 | 7958895 | 1.458 | Down | 3.51E-07 | Not Curated | Curated | https://www.ncbi.nlm.nih.gov/pmc/articles/PMC4030419/ |
| OLFM4 | 7969288 | 1.624 | Up | 2.38E-06 | Not Curated | Not Curated | https://www.ajog.org/article/S0002-9378(12)01599-2/pdf |
| RHD | 7898998 | 1.428 | Up | 2.75E-04 | Not Curated | Not Curated | Not Reported |
| RNASE3 | 7973105 | 1.29 | Up | 4.80E-04 | Not Curated | Curated | Not Reported |
| RNF182 | 8116980 | 0.6545 | Down | 3.24E-05 | Not Curated | Curated | Not Reported |
| RSAD2 | 8040080 | 0.8035 | Down | 1.54E-09 | Not Curated | Curated | http://amp.pharm.mssm.edu/Harmonizome/gene_set/Premature+Birth/CTD+Gene-Disease+Associations |
| SIGLEC14 | 8038885 | 0.9545 | Down | 6.62E-05 | Not Curated | Not Curated | Not Reported |
| SNORD116-1 | 7981949 | 0.8471 | Down | 3.08E-04 | Not Curated | Not Curated | Not Reported |
| SVBP | 7915468 | 0.6228 | Down | 2.37E-04 | Not Curated | Not Curated | Not Reported |
| TARP | 8139128 | 1.212 | Up | 2.20E-05 | Not Curated | Curated | Not Reported |
| TMEM176A | 8137264 | 0.9342 | Down | 3.39E-08 | https://bmcpregnancychildbirth.biomedcentral.com/articles/10.1186/1471-2393-9-56 | Curated | Not Reported |
| TMTC1 | 7962058 | 1.349 | Up | 1.58E-09 | https://bmcpregnancychildbirth.biomedcentral.com/articles/10.1186/1471-2393-9-56 | Not Curated | http://amp.pharm.mssm.edu/Harmonizome/gene_set/Premature+Birth/CTD+Gene-Disease+Associations |
| TUBB2A | 8116653 | 1.697 | Up | 8.79E-05 | Not Curated | Curated | Not Reported |
| USP32P1 | 8005225 | 0.7964 | Down | 5.02E-04 | Not Curated | Not Curated | Not Reported |
| UTS2 | 7912136 | 2.059 | Up | 2.31E-05 | Not Curated | Curated | Not Reported |
| VNN1 | 8129618 | 1.472 | Up | 9.31E-05 | Not Curated | Curated | https://journals.plos.org/plosone/article?id=10.1371/journal.pone.0155191 |

| **GO Term Name** | **Enriched Term ID** | **Corrected P-Value for enrichment** | **Number of Genes in the sPTB module mapped to the interactome** | **Number of genes enriched in each GO term** |
| --- | --- | --- | --- | --- |
| immune system process | GO:0002376 | 1.79E-11 | 36 | 28 |
| immune effector process | GO:0002252 | 7.95E-10 | 36 | 19 |
| leukocyte mediated immunity | GO:0002443 | 5.79E-09 | 36 | 16 |
| neutrophil degranulation | GO:0043312 | 9.97E-09 | 36 | 13 |
| neutrophil activation involved in immune response | GO:0002283 | 1.08E-08 | 36 | 13 |
| neutrophil activation | GO:0042119 | 1.43E-08 | 36 | 13 |
| neutrophil mediated immunity | GO:0002446 | 1.43E-08 | 36 | 13 |
| granulocyte activation | GO:0036230 | 1.66E-08 | 36 | 13 |
| regulated exocytosis | GO:0045055 | 2.17E-08 | 36 | 15 |
| immune response | GO:0006955 | 2.75E-08 | 36 | 22 |
| leukocyte degranulation | GO:0043299 | 3.19E-08 | 36 | 13 |
| myeloid cell activation involved in immune response | GO:0002275 | 4.02E-08 | 36 | 13 |
| myeloid leukocyte mediated immunity | GO:0002444 | 4.72E-08 | 36 | 13 |
| exocytosis | GO:0006887 | 1.46E-07 | 36 | 15 |
| myeloid leukocyte activation | GO:0002274 | 4.15E-07 | 36 | 13 |
| leukocyte activation involved in immune response | GO:0002366 | 1.01E-06 | 36 | 13 |
| cell activation involved in immune response | GO:0002263 | 1.08E-06 | 36 | 13 |
| cell activation | GO:0001775 | 1.14E-06 | 36 | 17 |
| secretion by cell | GO:0032940 | 2.99E-06 | 36 | 17 |
| export from cell | GO:0140352 | 4.69E-06 | 36 | 17 |
| defense response to other organism | GO:0098542 | 6.89E-06 | 36 | 15 |
| secretion | GO:0046903 | 1.09E-05 | 36 | 17 |
| defense response | GO:0006952 | 2.10E-05 | 36 | 17 |
| response to other organism | GO:0051707 | 2.42E-05 | 36 | 16 |
| response to external biotic stimulus | GO:0043207 | 2.47E-05 | 36 | 16 |
| response to biotic stimulus | GO:0009607 | 3.05E-05 | 36 | 16 |
| vesicle-mediated transport | GO:0016192 | 5.69E-05 | 36 | 18 |
| leukocyte activation | GO:0045321 | 0.000183 | 36 | 14 |
| innate immune response | GO:0045087 | 0.000668 | 36 | 12 |
| response to external stimulus | GO:0009605 | 0.000956 | 36 | 19 |
| response to stress | GO:0006950 | 0.010187 | 36 | 21 |
| multi-organism process | GO:0051704 | 0.011603 | 36 | 18 |
| secretory granule | GO:0030141 | 2.29E-09 | 36 | 15 |
| specific granule | GO:0042581 | 2.82E-09 | 36 | 9 |
| secretory vesicle | GO:0099503 | 2.97E-08 | 36 | 15 |
| tertiary granule | GO:0070820 | 1.45E-07 | 36 | 8 |
| specific granule lumen | GO:0035580 | 3.61E-07 | 36 | 6 |
| cytoplasmic vesicle part | GO:0044433 | 8.97E-07 | 36 | 16 |
| secretory granule membrane | GO:0030667 | 1.60E-05 | 36 | 8 |
| cytoplasmic vesicle | GO:0031410 | 0.000442 | 36 | 16 |
| intracellular vesicle | GO:0097708 | 0.00045 | 36 | 16 |
| secretory granule lumen | GO:0034774 | 0.000456 | 36 | 7 |
| cytoplasmic vesicle lumen | GO:0060205 | 0.000496 | 36 | 7 |
| vesicle lumen | GO:0031983 | 0.000516 | 36 | 7 |
| tertiary granule lumen | GO:1904724 | 0.000797 | 36 | 4 |
| tertiary granule membrane | GO:0070821 | 0.002349 | 36 | 4 |
| vesicle | GO:0031982 | 0.004522 | 36 | 19 |
| cytoplasmic vesicle membrane | GO:0030659 | 0.019504 | 36 | 8 |
| extracellular space | GO:0005615 | 0.021542 | 36 | 17 |
| vesicle membrane | GO:0012506 | 0.023488 | 36 | 8 |
| blood microparticle | GO:0072562 | 0.032803 | 36 | 4 |
| extracellular region | GO:0005576 | 0.042036 | 36 | 19 |
| whole membrane | GO:0098805 | 0.044581 | 36 | 11 |
| extracellular region part | GO:0044421 | 0.045204 | 36 | 17 |
| azurophil granule | GO:0042582 | 0.04603 | 36 | 4 |
| primary lysosome | GO:0005766 | 0.04603 | 36 | 4 |

**Table B**

**Table C**

| **Term Name** | **Enriched**  **Term ID** | **Corrected P-Value for enrichment** | **Number of Genes in the LCC of sPTB module mapped to the interactome** | **Number of genes enriched in each GO term** |
| --- | --- | --- | --- | --- |
| regulated exocytosis | GO:0045055 | 9.41E-14 | 20 | 15 |
| neutrophil degranulation | GO:0043312 | 3.98E-13 | 20 | 13 |
| leukocyte mediated immunity | GO:0002443 | 4.09E-13 | 20 | 15 |
| neutrophil activation involved in immune response | GO:0002283 | 4.31E-13 | 20 | 13 |
| neutrophil activation | GO:0042119 | 5.76E-13 | 20 | 13 |
| neutrophil mediated immunity | GO:0002446 | 5.76E-13 | 20 | 13 |
| granulocyte activation | GO:0036230 | 6.73E-13 | 20 | 13 |
| exocytosis | GO:0006887 | 6.97E-13 | 20 | 15 |
| leukocyte degranulation | GO:0043299 | 1.32E-12 | 20 | 13 |
| myeloid cell activation involved in immune response | GO:0002275 | 1.68E-12 | 20 | 13 |
| myeloid leukocyte mediated immunity | GO:0002444 | 1.99E-12 | 20 | 13 |
| immune system process | GO:0002376 | 2.04E-12 | 20 | 20 |
| myeloid leukocyte activation | GO:0002274 | 1.91E-11 | 20 | 13 |
| cell activation | GO:0001775 | 2.08E-11 | 20 | 16 |
| leukocyte activation involved in immune response | GO:0002366 | 4.85E-11 | 20 | 13 |
| cell activation involved in immune response | GO:0002263 | 5.22E-11 | 20 | 13 |
| immune effector process | GO:0002252 | 1.07E-10 | 20 | 15 |
| secretion by cell | GO:0032940 | 1.91E-09 | 20 | 15 |
| export from cell | GO:0140352 | 2.92E-09 | 20 | 15 |
| secretion | GO:0046903 | 6.57E-09 | 20 | 15 |
| vesicle-mediated transport | GO:0016192 | 9.11E-09 | 20 | 16 |
| leukocyte activation | GO:0045321 | 1.18E-07 | 20 | 13 |
| immune response | GO:0006955 | 5.39E-07 | 20 | 15 |
| defense response to other organism | GO:0098542 | 0.000292 | 20 | 10 |
| innate immune response | GO:0045087 | 0.000839 | 20 | 9 |
| defense response | GO:0006952 | 0.001003 | 20 | 11 |
| response to other organism | GO:0051707 | 0.002982 | 20 | 10 |
| response to external biotic stimulus | GO:0043207 | 0.003018 | 20 | 10 |
| response to biotic stimulus | GO:0009607 | 0.003459 | 20 | 10 |
| transport | GO:0006810 | 0.007801 | 20 | 16 |
| establishment of localization | GO:0051234 | 0.010872 | 20 | 16 |
| response to stress | GO:0006950 | 0.016417 | 20 | 14 |
| negative regulation of lymphocyte mediated immunity | GO:0002707 | 0.016503 | 20 | 3 |
| response to external stimulus | GO:0009605 | 0.018315 | 20 | 12 |
| multi-organism process | GO:0051704 | 0.031862 | 20 | 12 |
| negative regulation of leukocyte mediated immunity | GO:0002704 | 0.033594 | 20 | 3 |
| localization | GO:0051179 | 0.039275 | 20 | 17 |
| secretory granule | GO:0030141 | 1.04E-14 | 20 | 15 |
| secretory vesicle | GO:0099503 | 1.55E-13 | 20 | 15 |
| specific granule | GO:0042581 | 4.74E-12 | 20 | 9 |
| cytoplasmic vesicle part | GO:0044433 | 6.43E-11 | 20 | 15 |
| tertiary granule | GO:0070820 | 5.70E-10 | 20 | 8 |
| specific granule lumen | GO:0035580 | 6.27E-09 | 20 | 6 |
| cytoplasmic vesicle | GO:0031410 | 4.04E-08 | 20 | 15 |
| intracellular vesicle | GO:0097708 | 4.11E-08 | 20 | 15 |
| secretory granule membrane | GO:0030667 | 6.94E-08 | 20 | 8 |
| secretory granule lumen | GO:0034774 | 4.49E-06 | 20 | 7 |
| cytoplasmic vesicle lumen | GO:0060205 | 4.89E-06 | 20 | 7 |
| vesicle lumen | GO:0031983 | 5.1E-06 | 20 | 7 |
| vesicle | GO:0031982 | 5.16E-05 | 20 | 15 |
| tertiary granule lumen | GO:1904724 | 5.69E-05 | 20 | 4 |
| cytoplasmic vesicle membrane | GO:0030659 | 0.000122 | 20 | 8 |
| vesicle membrane | GO:0012506 | 0.00015 | 20 | 8 |
| tertiary granule membrane | GO:0070821 | 0.00017 | 20 | 4 |
| endomembrane system | GO:0012505 | 0.000368 | 20 | 15 |
| azurophil granule | GO:0042582 | 0.003516 | 20 | 4 |
| primary lysosome | GO:0005766 | 0.003516 | 20 | 4 |
| whole membrane | GO:0098805 | 0.004374 | 20 | 9 |
| specific granule membrane | GO:0035579 | 0.021212 | 20 | 3 |
| bounding membrane of organelle | GO:0098588 | 0.02332 | 20 | 9 |
| platelet alpha granule membrane | GO:0031092 | 0.027002 | 20 | 2 |
